# Splenic torsion mistaken for an ovarian cyst: a case report 

**DOI:** 10.1186/s13256-024-04502-6

**Published:** 2024-04-14

**Authors:** Salaar Ahmed, Shahid Iqbal, Shariqa Batool, Rizwan Khan

**Affiliations:** 1 Medical College, Aga Khan University, Stadium Road, Karachi, 74800 Pakistan; 2 Department of Surgery, Aga Khan University, Karachi, Pakistan

**Keywords:** Wandering spleen, Ovarian cyst, Splenic torsion, Splenectomy

## Abstract

**Background:**

Wandering spleen (or ectopic spleen) refers to a hyper-mobile spleen resulting in its displacement from the normal anatomical position to usually in the lower abdominal or pelvic cavity. While ultrasound is often the first radiological modality used, Computed Tomography (CT) shows a clear picture and aides to reach a diagnosis. In circumstances where appropriate imaging modalities are not available, or the operator is inexperienced, diagnosis of wandering spleen can be missed.

**Case presentation:**

A 22-nulligravida unmarried Sindhi female had presented to the Emergency Room (ER) with a 5-day history of intermittent severe lower abdominal pain. An ultrasound at a local practitioner had suggested an ovarian cyst. Ultrasound-pelvis and later CT scan at our facility reported an enlarged wandering spleen with torsion of its pedicle and infarction. Exploratory laparotomy with splenectomy was done. An enlarged wandering spleen was found with torsion of the splenic vein and thrombosed arterial supply from omentum wrapped over the mass. The patient developed thrombocytosis post-surgery but otherwise did well and was discharged after 2 days.

**Conclusion:**

Splenic torsion secondary to a wandering spleen can be challenging to diagnose, especially in resource limited settings where ultrasound might be the only modality available. Timely diagnosis and proper intervention are key to saving the life and the spleen.

## Background

Wandering spleen (or ectopic spleen) refers to a hyper-mobile spleen resulting in its displacement from the normal anatomical position to usually in the lower abdominal or pelvic cavity. In most rural settings, ultrasound is often the first radiological modality used since it’s inexpensive and portable. In contrast, Computed Tomography (CT) scan can provide a more detailed picture, helping to differentiate a case of splenic torsion in a wandering spleen from other causes of lower abdominal pain (such as ovarian cyst in our case). In circumstances where appropriate imaging modalities are not available, or the operator is inexperienced, diagnosis of this condition can be missed, which can sometimes lead to fatal consequences.

## Case presentation

A 22-year-old nulligravida, unmarried Sindhi female presented to the Emergency Room (ER) with a 5-day history of intermittent, severe lower abdominal pain on April 12th, 2023. The patient complained of throbbing, non-radiating pain. Initially, the patient had visited her local practitioner, who had advised an abdominal and pelvic ultrasound. The ultrasound revealed an oval hypoechoic area in the right lower abdomen measuring 15.9 × 8.8 × 5.9 cm and showing no blood flow on color Doppler. The report was suggestive of a right ovarian cyst. The local practitioner had thus referred her to our facility for drainage of the cyst.

At the time of presentation, the patient complained of mild pain in the lower abdomen but was otherwise vitally stable. Her medical, surgical, and menstrual histories were all unremarkable. On GPE, the abdomen was soft and non-tender with a hard mass in the right hypochondrium.

On the day of admission, she was anemic with a hemoglobin count of 9 g/dl. She also had leukocytosis (WBC = 15.2), with predominant neutrophilia, and a normal platelet count. Her Liver Function Tests (LFTs) were deranged with elevated direct bilirubin (0.7), alkaline phosphate (184), and LDH (315). Tests for various tumor markers showed an increased serum CA-125 (351 IU/ml), while serum Beta HCG, serum alpha-fetoprotein, and CEA were in the normal range.

**Fig. 1:**
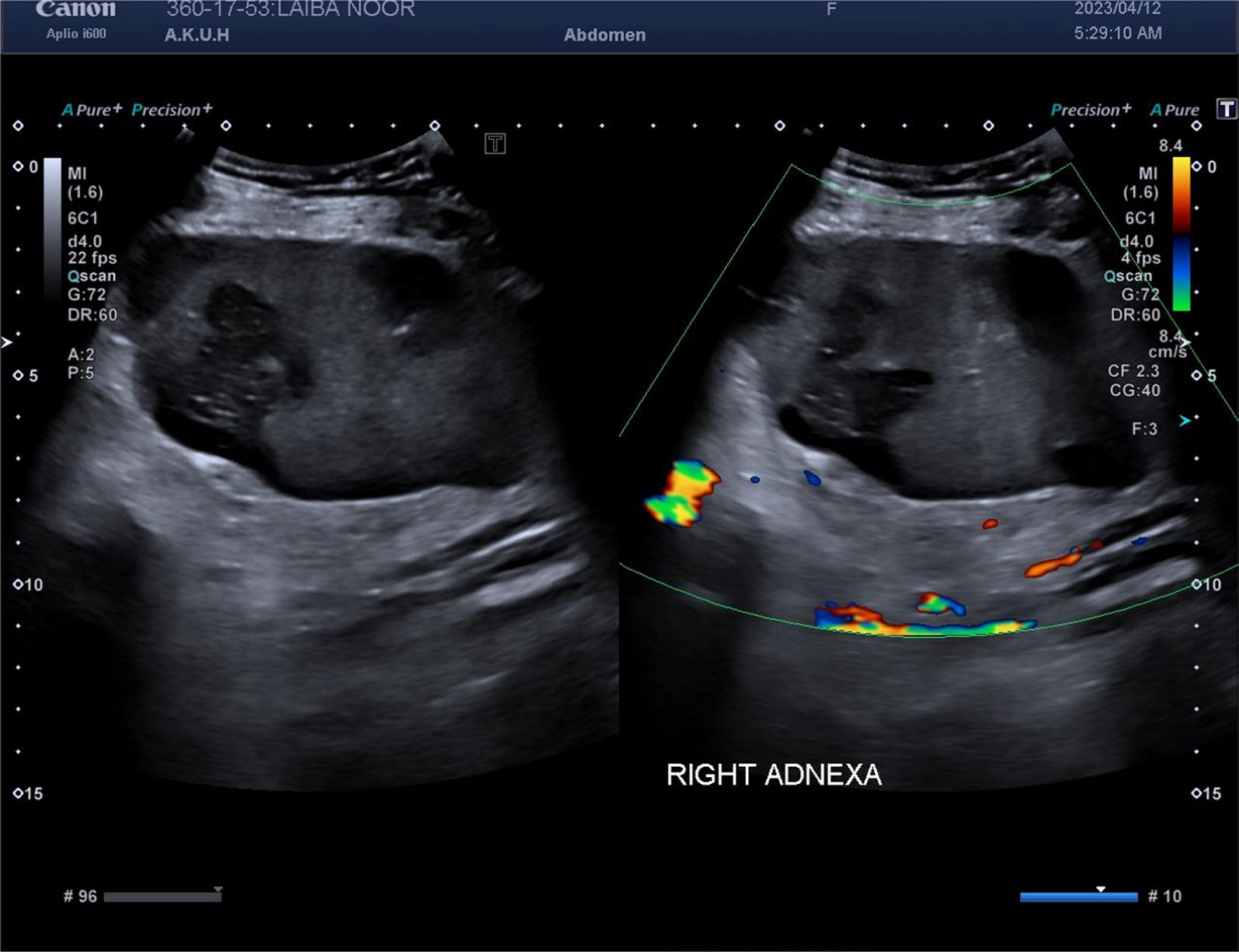
Ultrasound of right pelvis. A solid cum cystic mass with specs of calcification can be noted

**Fig. 2:**
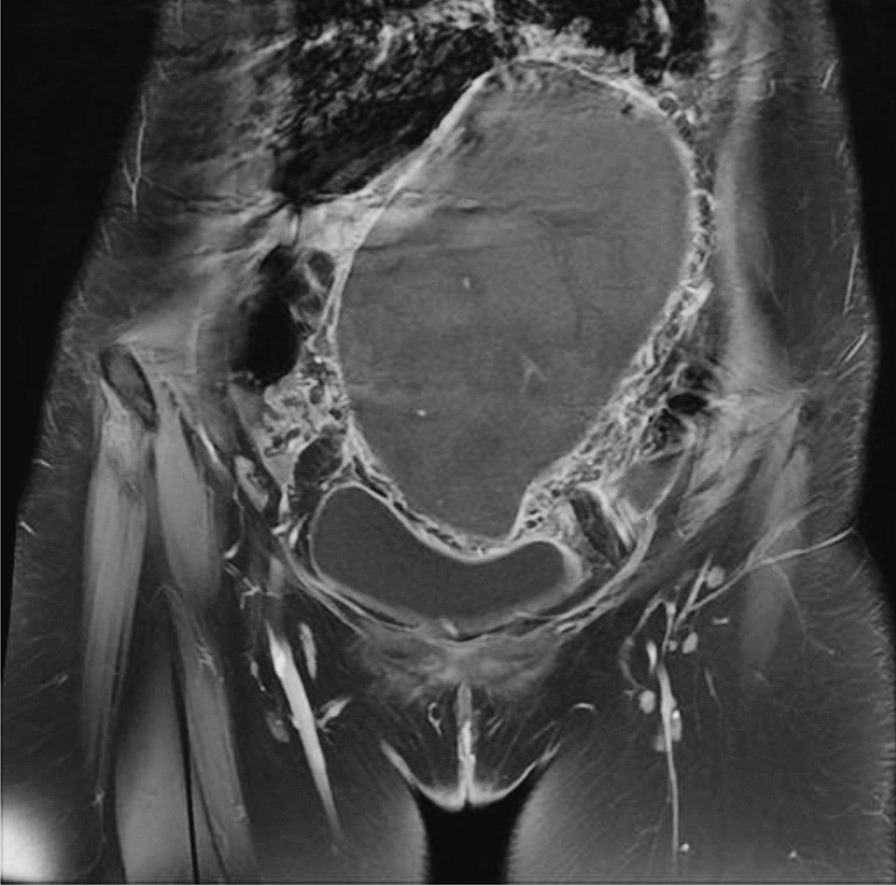
MRI pelvis showing a large obliquely placed mass. Swirling of the vessels can be noted on the left side

Ultrasound-pelvis at our facility reported a solid cum cystic heterogeneous mass with specks of calcification in the right periumbilical region measuring 155 × 101 × 55 mm (Fig. 1). Based on the indefinite ultrasound findings, an MRI of the pelvis was ordered, which showed a 164 × 105 mm obliquely placed comma-shaped mass in the lower abdomen/pelvic region (Fig. 2). Twisting and swirling of vessels was noted on the left side that extended into the mass. The ovaries, however, were normal in size. The radiology findings were suggestive of an enlarged wandering spleen with the torsion of its pedicle and infarction.

**Fig. 3:**
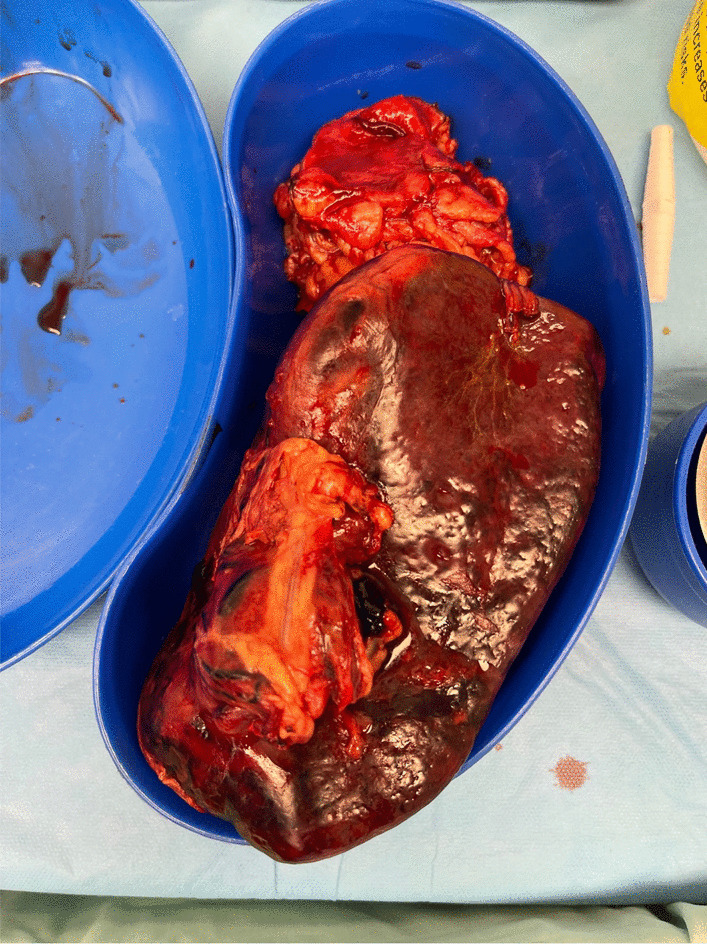
Enlarged and infarcted spleen specimen obtained after splenectomy. Note that the spleen was 164 * 105 mm in length, which is twice the size of normal spleen

An exploratory laparotomy with a possible splenectomy was advised and eventually performed. A midline lower abdominal incision was given to reach the peritoneal cavity. An enlarged wandering spleen was found with torsion of the splenic vein and thrombosed arterial supply from omentum wrapped over the mass. A splenectomy was performed, and a tissue cross-section was sent for histopathology that later reported hemorrhagic infarction and congestion of the spleen and separate omental tissue (Fig. 3). There were no signs of any malignancy.

Postoperatively the patient was clinically and vitally stable. She had two episodes of vomiting on the second post-op day (April 15th), which were managed with ondansetron. The Hemoglobin and WBC count were in the normal range, but her platelets remained high throughout her post-operative stay. She recovered well and was discharged 2 days later on April 17th, 2023.

## Discussion

Wandering spleen (or ectopic spleen) refers to a hyper-mobile spleen resulting in its displacement from normal anatomical position in the left hypochondrium to an ectopic site usually in the lower abdominal or the pelvic cavity [1, 2]. Considered a rare clinical presentation with an overall incidence rate of less than 0.2%, it has been reported mostly in children within the first decade of life. For adults, most literature exists on young females (such as our patient), who are multiparous (probability of 0.7–0.8 cases between 20 and 40 years of age) compared to males with a ratio of 7:1 [3, 4].

Patients often present as asymptomatic and thus it is usually an accidental finding on a radiographic scan, but some may present with complaints of intermittent abdominal discomfort or palpable abdominal mass on examination as was observed in our patient. In symptomatic cases, acute or chronic abdominal pain is secondary to splenic enlargement while more emergent conditions include splenic rupture, hemoperitoneum, and peritonitis [5, 6]. These mostly correlate with either intermittent torsion and impulsive detorsion of splenic arteries and veins, splenic infarction, gangrene, abscess or splenic fibrosis/necrosis, pancreatitis, gastric volvulus, and gastric variceal bleeding [3, 7]. Our patient was initially misdiagnosed as Doppler ultrasound reports delineated an oval hypoechoic mass in the mid, lower right abdomen which was interpreted as an ovarian cyst, and a cyst drainage was advised. While an ovarian cyst and wandering spleen may be difficult to differentiate solely via ultrasound, a wandering spleen can be included as a differential in young females presenting with a hard mass in the right hypochondrium palpable up to the umbilicus, restricted motility, and unremarkable menstrual history or hormonal changes.

The subsequent diminished blood flow to the spleen results in RBC and platelet sequestration, leading to anemia and thrombocytopenia. While being anemic with a hemoglobin count of 9 g/dl, our patient in contrast was suffering from neutrophilic leukocytosis due to hypoxemia, tissue necrosis, and possible secondary bacterial infection. Additionally, deranged liver enzymes such as elevated LDH and alkaline phosphatase were recorded which may be due to possible portal vein congestion secondary to obstruction of splenic vessels [8].

Torsion of splenic pedicles lies within the range of 90°–2160°. The degree of splenic torsion determines either partial or complete splenic infarction with or without splenic rupture. The torsion can also be classified as either acute or chronic with acute, imitating the symptoms of appendicitis, bowel obstruction, or twisted ovarian cysts whereas the chronic form emulates an abdominal mass present in any of the four quadrants [9]. In our case, the torsion must have been at least 720° as torsion of splenic veins was also detected, while the arterial supply was thrombosed and wrapped over the spleen from the omentum.

The patient primarily presented with complaints of intermittent abdominal pain but no signs of vomiting, constipation, or abdominal distension. She was nulliparous whereas pregnancy and multiparity are the prominent predisposing factors leading to diagnosis of WS due to hormonal changes, laxity/lengthening of splenic ligaments, and/or weakness of abdominal wall. The patient did not have apparent symptoms indicating peritonitis or condition of shock signifying possible hemoperitoneum, both of which are possible complications of splenic pedicle torsion as seen in our case. This can be explained by the fact that hemorrhagic infarction and splenic congestion were present, but no splenic rupture was discernible.

Thrombocytopenia can also be present in rare cases of splenic torsion while, the patient in our case on the contrary, had a normal-high platelet count both pre and post operative which can be explained by post splenectomy reactive thrombocytosis. The condition is benign and self-resolving in the majority of cases [10]. Moreover, the patient had an elevated serum tumor marker CA-125 and a right adrenal neoplastic mass but no malignancy was reported on histopathological examination which was later confirmed as the dislocated spleen. Hence the clinical presentation in our case differs greatly from the clinical presentation of a typical patient with WS.

While ultrasound is often the first radiological modality used in most cases, multi-slice spiral CT is more accurate, especially for early detection. Moreover, CT clearly establishes the heterogeneity or homogeneity of the spleen parenchyma and the organ’s arterial and venous vascularization [11–13]. Since a Doppler ultrasound, often, reveals a heterogenous, hypoechoic mass with specks of calcifications in the periumbilical region as noted in our patient, this could be confused for an ovarian cyst, especially if the radiologist performing the U/S does not have this differential in mind.

Furthermore, the CT contrast findings of the patient corroborated bilateral dense breast parenchyma with two homogenous and well-defined lesions in the left breast. For this concern, the patient was given a referral to visit a breast surgeon for evaluation and further investigation, but the patient did not follow up.

Surgery which includes splenectomy (open or laparoscopically) or splenopexy is the optimal standard of treatment. A laparoscopic splenopexy is the most practiced approach where splenic preservation is necessary (usually under 30 years of age) [14]. Splenectomy is a preferable choice in conditions of splenic infarction, hypersplenism, or splenomegaly [2, 15]. Our patient presented with pedicle torsion and splenic infarction hence, a splenectomy was performed.

All patients are usually advised to receive *H. influenzae*, pneumococcus, and meningococcus vaccines post-splenectomy as was prescribed and administered to our patient post-operation to avoid post-splenectomy sepsis, as the risk of infection and chronic disease is higher in such patients and can lead to adverse outcomes [16]. Our patient was also advised to follow up with her primary care physician for vaccination.

## Conclusion

Splenic torsion secondary to a wandering spleen can be a challenging diagnosis to make for both a clinician and radiologist. The timely identification of this rare condition, immediate life-saving measures, and prompt surgical intervention are crucial in preserving the spleen, if possible, and avoiding potentially fatal complications.

## Data Availability

All data underlying the results are available as part of the article and no additional data sources are required.

## Availability of supporting data

Authors have full intellectual ownership of the article and agree to the data-sharing policy of the Journal.
